# Reversible Cerebral Vasoconstriction Syndrome Secondary to Paroxetine and Opioid Use in a Middle-Aged Woman: A Case Report

**DOI:** 10.7759/cureus.79917

**Published:** 2025-03-02

**Authors:** Evan Simonsen, Dana N Joseph, Newlyn Joseph

**Affiliations:** 1 Department of Psychiatry, Albany Medical Center, Albany, USA; 2 Department of Psychiatry, Geisel School of Medicine at Dartmouth, Hanover, USA

**Keywords:** neurological outcomes, opioid use disorders, paroxetine, past psychiatric history and depression, reversible cerebral vasoconstriction syndrome (rcvs), selective serotonin reuptake inhibitor (ssri)

## Abstract

Reversible cerebral vasoconstriction syndrome (RCVS) is a condition that usually presents with recurrent thunderclap headaches, focal neurological deficits, and seizures, secondary to transient cerebral vascular spasms. Although the mechanism is not well understood, medications that modulate serotonergic activity, such as selective serotonin reuptake inhibitors (SSRIs), may induce RCVS. In this case, we present a 39-year-old female patient with thunderclap headaches and a multitude of neurological deficits, including vision loss and musculoskeletal weakness. Her medical history is significant for depression, migraines, and opioid-use disorder in remission, treated with paroxetine and mirtazapine, sumatriptan, and buprenorphine-naloxone, respectively. The diagnosis of RCVS was supported by imaging that demonstrated multiple acute cerebral infarcts and stenoses. The patient spent a total of eight days as an inpatient, and during that time, her symptoms showed gradual improvement. RCVS is a rare condition that should be considered in a patient with risk factors for whom high-acuity diagnoses such as subarachnoid hemorrhage have been ruled out.

## Introduction

Reversible cerebral vasoconstriction syndrome (RCVS) is characterized by transient constriction of cerebral arteries, leading to symptoms such as thunderclap headaches, focal neurological deficits, and seizures. The condition is typically reversible within three months, and the prognosis is generally favorable with appropriate management. However, complications such as ischemic stroke or cerebral hemorrhage can occur if not promptly diagnosed and treated [1]. Paroxetine is a selective serotonin reuptake inhibitor (SSRI) commonly prescribed for various psychiatric conditions, including depression and anxiety disorders. While effective for these indications, paroxetine has been associated with several side effects, including the rare induction of RCVS. The mechanism by which SSRIs like paroxetine might induce RCVS is not fully understood but is thought to involve alterations in serotonergic regulation of cerebral vascular tone [2].

Opioid use disorder is a chronic relapsing condition characterized by the compulsive use of opioids despite adverse consequences. Management includes a combination of medication-assisted treatment, counseling, and support groups [3]. Patients with opioid use disorder may be at increased risk for psychiatric comorbidities, necessitating the use of SSRIs like paroxetine [4]. Some opioids can influence serotonergic pathways [5], and their interaction with SSRIs like paroxetine may increase the risk of adverse events such as RCVS.

In this case, a patient with opioid use disorder presents with acute symptoms consistent with RCSV after adjustment of paroxetine dose.

## Case presentation

A 39-year-old female patient presented as a transfer from another hospital with a 30-day history of constant, pulsating headache with acutely worsening symptoms. She described her headache becoming acutely worse with a “pop” seven days prior to her presentation to the hospital. She endorsed photophobia and phonophobia, transient bilateral lower extremity weakness and numbness, and four to five days of bilateral vision loss, with only the right upper quadrant being preserved. Past medical history is significant for hypertension, depression, migraines, and a 10-year history of opioid use disorder in remission. She has been taking lisinopril 10 milligrams daily, paroxetine 40 milligrams daily, increased from 30 milligrams daily approximately three months prior to presentation, mirtazapine 30 milligrams daily, buprenorphine-naloxone 8-2 milligrams twice daily for nine years, and sumatriptan 25 milligrams as needed. Social history was unremarkable for alcohol or recent illicit drug use; the patient had abused oral prescription opioids for 10 years but had not had any relapses since starting buprenorphine.

**Figure 1:**
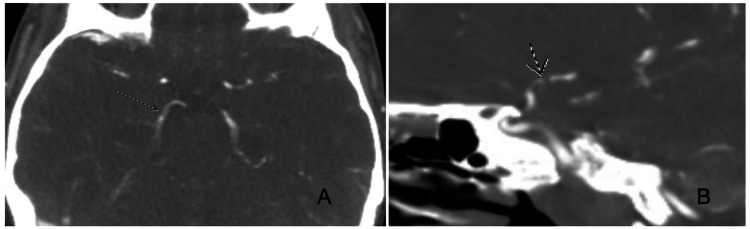
CT angiography (CTA) head demonstrating right posterior cerebral artery (PCA) multifocal stenoses throughout its course in the (A) axial section, including a moderate to high-grade stenosis within the proximal P1 segment, which is more appreciable in the (B) sagittal plane. These findings are consistent with reversible cerebral vasoconstriction syndrome (RCVS), a condition characterized by the transient narrowing of cerebral arteries. Stenosis is hypothesized to result from multiple pathophysiological mechanisms, including endothelial dysfunction, sensitivity to vasoactive substances, and sympathetic nervous system involvement.

**Figure 2:**
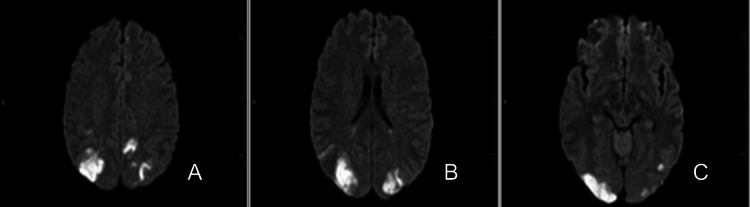
MRI brain (diffusion-weighted imaging (DWI) series, axial sections) demonstrating acute/subacute multifocal infarcts in a multi-arterial territory distribution, including the (A) bilateral parietal lobes and (B, C) bilateral occipital lobes. These findings are suggestive of reversible cerebral vasoconstriction syndrome (RCVS), a condition that can lead to cerebral ischemia due to the transient narrowing of multiple cerebral arteries. The infarcts observed in distinct arterial territories are a common manifestation of RCVS, which is often associated with sudden-onset headaches and can result in neurological deficits, including vision impairments, as seen in this patient.

The physical exam was significant for blurred vision, with the patient unable to determine how many fingers the examiner held up in each quadrant. Vital signs were significant for mild tachycardia and hypertension with a blood pressure of 161/104. The discharge paperwork from the outside hospital noted that her hospital course was complicated by bradycardia in the 20s, which appeared to occur when she was sleeping. Laboratory tests, including a tick panel, Lyme total antibodies (ABS), cerebrospinal fluid (CSF) Lyme, perinuclear anti-neutrophil cytoplasmic antibody (p-ANCA), Sjögren’s syndrome-related antigen A (SS-A/Ro) and antigen B (SS-B/La) antibodies, double-stranded DNA (dsDNA), smooth muscle antibody, ribonucleoprotein (RNP) antibody, and anti-cardiolipin immunoglobulin G (IgG) antibody, were unremarkable. Imaging performed at the sending facility included a computed tomography (CT) of the head, as well as CT angiography, that demonstrated multiple patchy hypodensities in both cerebral hemispheres and extensive multifocal stenosis throughout the cerebral arterial circulation bilaterally, most prominently in the right posterior cerebral artery (PCA) (Figure 1). Magnetic resonance imaging of the brain with and without contrast demonstrated acute/subacute multifocal infarcts in multiple cerebral vascular territories (Figure 2). A CT angiogram of the neck showed no evidence of steno-occlusive changes or dissection. A lumbar puncture performed at the outside hospital was also unremarkable.

The patient was admitted to the neurological intensive care unit (ICU) and started on atorvastatin 80 milligrams and aspirin 81 milligrams daily for secondary stroke prevention, as well as verapamil 40 milligrams three times daily for headaches. She was seen by neurology, ophthalmology, psychiatry, and neurosurgery and spent a total of eight days on the inpatient floor. Her presentation was concerning for vasculitis versus RCVS, the latter of which was ultimately diagnosed following an angiogram on hospital day 7. Her visual acuity and muscle weakness gradually improved over the course of her hospital stay, although severe deficits remained. At the time of discharge, the patient was beginning to regain vision in the right lower quadrant, but still lacked a response to threat testing bilaterally. She was discharged with instructions to follow up with neurosurgery and ophthalmology, as well as begin physical rehabilitation for her visual and motor deficits. She was also encouraged to start outpatient cognitive behavioral therapy to address her new diagnosis of adjustment disorder with anxiety. Lastly, as her serotonergic medications were stopped during her hospital course, she was advised to follow up with outpatient psychiatry to determine a new medication regimen for her depression.

## Discussion

RCVS is a condition characterized by a new-onset thunderclap headache with accompanying focal neurological symptoms or seizures. RCVS is diagnosed based on key clinical features of thunderclap headache (reaching peak intensity within ≤1 minute) or severe recurrent headache, cerebral vasoconstriction on imaging in at least two different arteries and resolution of vasoconstriction by three months, in the absence of primary angiitis of the central nervous system (PACNS), or aneurysmal subarachnoid hemorrhage (SAH) [6]. Although the pathophysiology of RCVS is not fully understood, a confluence of factors, including abnormal cerebrovascular tone, endothelial dysfunction, sympathetic hyperstimulation, and oxidative stress, appear to be implicated in the syndrome [7].

The RCVS2 score is a validated tool that can also be used to support the diagnosis of RCVS [8]. This tool assigns a varying number of points for clinical and historical features, including recurrent or single thunderclap headache, intracranial carotid artery involvement, identification of a vasoconstrictive trigger, sex, and the presence of SAH on imaging. A score equal to or less than 2 indicates a likely negative diagnosis, while a score equal to or greater than 5 indicates a likely positive diagnosis. Scores of 3 or 4 are considered equivocal. This patient’s RCVS2 score was 9, which supported the diagnosis of RCVS.

Most cases occur either postpartum or after exposure to either adrenergic or serotonergic drugs [9]. Although RCVS has been increasingly recognized in the past two decades, most of the existing literature describes the association between RCVS and SSRIs through case reports. The FDA Adverse Event Reporting System (FAERS) Public Dashboard shows that a total of 29 cases of paroxetine-associated RCVS have been reported to the FDA.

While no randomized controlled clinical trials exist to provide guidance on the best treatment for RCVS, observational studies suggest that verapamil is a reasonable treatment option [10]. Because RCVS is predominantly self-limited, current treatment approaches generally focus on symptomatic management of headaches and hyperglycemia, which may worsen outcomes [7]. Importance is placed on avoiding misdiagnosis, which may lead to more invasive treatment and diagnostic approaches that could potentially worsen patient outcomes as well.

## Conclusions

In conclusion, this case highlights a rare presentation of RCVS in a middle-aged female with a complex psychiatric and medical history, including depression, migraine, and opioid use disorder in the setting of paroxetine dose escalation and chronic buprenorphine-naloxone use. Although the link between opioid use and RCVS is not as firmly established as the link between RCVS and SSRIs, it still warrants careful medication review when treating patients with these comorbidities. Caution should be exercised when prescribing opioids to patients with cerebrovascular conditions suggestive of RCVS with concomitant serotonergic medication use. While RCVS is a relatively uncommon condition, this case underscores the importance of careful medication review and contributes to the growing body of literature associating SSRI use with RCVS.
